# A SuperSelective primer-based real-time PCR platform for hypersensitive detection of azole resistance in *Cryptococcus neoformans*

**DOI:** 10.1128/spectrum.01166-26

**Published:** 2026-09-29

**Authors:** Siddhi Pawar, Honglin H. Xue, Sophia Wang, Salvatore A. E. Marras, Chaoyang Xue

**Affiliations:** 1 Public Health Research Institute, New Jersey Medical School, Rutgers University, Newark, New Jersey, USA; 2 Department of Microbiology, Biochemistry, and Molecular Genetics, New Jersey Medical School, Rutgers University, Newark, New Jersey, USA; University of Florida College of Dentistry, Gainesville, Florida, USA

**Keywords:** *Cryptococcus neoformans*, resistance, fluconazole, molecular beacon, SuperSelective primer

## Abstract

*Cryptococcus neoformans* is the leading cause of fungal meningitis with limited treatment options, making early and accurate diagnosis critical for improved patient outcomes. Current diagnostic methods for cryptococcosis rely largely on capsule antigen detection and fungal culture, which are time-consuming and unable to identify mutation-driven antifungal resistance. In this study, we have developed a SuperSelective primer-based PCR (SSP-PCR) platform for the rapid and specific detection of azole resistance-associated single-nucleotide polymorphisms (SNPs). We demonstrate that SSP-PCR reliably detected a single copy of mutant allele in the presence of excess wild-type (WT) DNA, with sensitivity reaching a 1:10^4^ mutant-to-WT ratio. Incorporating molecular beacon (MB) probes into our SSP-PCR platform further enhanced amplification specificity, enabling selective detection of the *ERG11* Y145F (A434T) mutation, which is known to confer high azole resistance. Using genomic DNA from *in vitro* cultures and mouse lung tissues infected with either WT strain H99 or a fluconazole-hyper-resistant *mrl1* clinical isolate that carries the *ERG11*(A434T) mutation, or both strains, we successfully detected the A434T mutant allele in both settings. Moreover, our SSP-PCR simultaneously identified *ERG11*(A434T) and the multi-azole resistance-associated *ERG11*(G1885A) mutant alleles in a single-tube duplex reaction. Collectively, this is the first study that utilizes the SSP-PCR platform to provide a robust and ultrasensitive molecular approach for the detection of azole resistance in *C. neoformans,* which provides a proof-of-concept methodology for its potential high-throughput clinical screening applications.

**IMPORTANCE:**

Invasive fungal infections are a growing public health threat causing over 1.5 million deaths annually, with cryptococcal meningitis accounting for over 15% of HIV/AIDS-related mortality. The problem is aggravated by limited treatment options and emerging drug resistance. Long-term use of fungistatic azoles like fluconazole promotes the emergence of azole resistance and heteroresistance, contributing to clinical treatment failure. Current diagnostic assays often fail to detect resistance-associated mutations within heterogeneous fungal populations, limiting their clinical utility. In this study, we developed a SuperSelective primer-based PCR (SSP-PCR) platform for the rapid and ultrasensitive detection of azole resistance-associated single-nucleotide polymorphisms (SNPs) in *Cryptococcus neoformans*. By integrating molecular beacon probes, this assay achieves high specificity and enables the detection of multiple SNP mutations in a single reaction. Our SSP-PCR platform offers a powerful molecular approach for identifying azole resistance with the potential to improve diagnostic precision and guide antifungal therapy in clinical settings.

## INTRODUCTION

Invasive fungal infections represent an increasing public health threat worldwide, causing over 1.5 million deaths annually (1–3). *Cryptococcus neoformans* (*Cn*) is an opportunistic fungal pathogen that causes infection through inhalation of spores and yeasts. Following pulmonary infection, *Cn* can disseminate to the central nervous system to cause life-threatening fungal meningitis, accounting for ~15% HIV/AIDS-related deaths globally (4). Despite these alarming mortality rates, therapeutic options remain limited, and the problem is aggravated by their collateral effects and drug resistance. Currently, polyenes and azoles are the backbone of anticryptococcal therapy. The polyene amphotericin B (AmB) is a rapidly acting fungicidal against *Cn*, albeit being highly toxic (5). Patients with cryptococcal meningitis are typically treated with AmB and 5-flucytosine first, then switched to triazoles, such as fluconazole (FLC), for long-term maintenance therapy. FLC is the preferred choice for maintenance therapy due to its low toxicity and oral bioactivity. However, long-term use of fungistatic azoles like FLC has allowed the fungus to persist in immunocompromised hosts. The repeated exposure to FLC enables the fungal isolates to mutate and develop resistance, resulting in suboptimal clinical outcomes (6, 7). The resistance often results in clinical treatment failure, and alterations in azole susceptibility in cryptococcal meningitis patients on long-term FLC maintenance therapy (8–12). Compounding this challenge, the lack of simple diagnostic methods to identify potential azole resistance and heteroresistance in clinical samples contributes to poor patient outcomes.

The current diagnosis of cryptococcosis relies on a combination of histopathology, direct microscopy, fungal culture, and detection of cryptococcal antigen (CrAg) in bodily fluids. Among these, antigen-based assays are widely used for their high sensitivity and high specificity (13). These assays detect capsular polysaccharide antigen in serum and cerebrospinal fluid. In clinical settings, commonly used CrAg detection methods include enzyme-linked immunosorbent assay (ELISA), latex agglutination, and lateral flow assay (LFA) (13). Of these, LFA is the most employed diagnostic approach in clinical settings because it is rapid, simple, and cost-effective. However, none of these methods can detect mutation-induced resistance.

Commonly used triazole drugs, such as fluconazole, target lanosterol 14-alpha-demethylase encoded by the *ERG11* gene located on chromosome 1 of the *Cn* genome. Azole resistance in *Cn* arises mostly through three mechanisms: genetic mutations in the *ERG11* gene, overexpression of *ERG11* due to aneuploidy caused by duplication of the chromosome one genomic region, and enhanced efflux pumps due to overexpression of transporter genes *AFR*1 and *MDR*1 (14). A single nucleotide mutation in *ERG11* can confer resistance (15, 16). Extensive analysis of clinical and environmental isolates of *Cn* and *C. gattii* by Dr. Kwon-Chung’s group revealed that the Y145F (A434T) single mutation in *ERG11* causes high-level FLC resistance (9), while the *ERG11*(G1885A) point mutation results in multi-azole resistance (17). Detecting resistance within a mixed fungal population remains extremely challenging and is not achievable with currently available diagnostic techniques.

In this study, we have developed a SuperSelective primer-based real-time PCR (SSP-PCR) platform for rapid and specific detection of azole resistance associated with single-nucleotide polymorphisms (SNPs) in the *Cn ERG11* gene, including *ERG11*(A434T) and *ERG11*(G1885A). The unique design of SuperSelective primers enables highly specific recognition and quantitative detection of mutant targets, even in the presence of closely related wild-type sequences (17–19). Upon hybridization to the target sequence, these primers initiate synthesis of mutant DNA fragments, generating amplicons that undergo exponential amplification in subsequent thermal cycles (18–20). To our knowledge, this is the first study to employ the SuperSelective primer design to detect azole-associated antifungal drug resistance mutations in fungi and *Cn*. Additionally, the incorporation of molecular beacons (MBs) enabled the development of a duplex real-time PCR assay that simultaneously detects two mutant target sequences in a single reaction. MBs are stem loop-shaped oligonucleotide probes that fluoresce upon hybridization to DNA or RNA target sequences (21, 22) and have been widely used to identify different human pathogens, including fungi (23). Collectively, our work established the first SSP-PCR platform for the rapid detection of azole-resistance-associated SNP mutations in *Cn*, validated *in vitro* using both synthetic g-Blocks and genomic DNA and *in vivo* infected tissues.

## RESULTS

### Designing SuperSelective primers for real-time PCR detection of *ERG11*-associated mutations

SuperSelective primers consist of three parts: (i) anchor sequence, (ii) bridge sequence, and (iii) foot sequence (Fig. 1A). The 5′-anchor sequence, like a conventional PCR primer, binds only to the DNA region of interest. The 3′-foot sequence is separated from the anchor by a bridge sequence that forms a single-stranded bubble in the resulting hybrid. The 3′-foot sequence is a short sequence that possesses an “interrogating nucleotide,” allowing it to form complementary hybrids with mutation-containing target sequence, resulting in the generation of amplicons (Fig. 1A). Following the design guidelines by Vargas et al. (17), we designed SuperSelective primers for detection of *Cn ERG11*(A434T) and *Cn ERG11*(G1885A) mutations that are known to cause high-level FLC resistance (Table 1). The SuperSelective primer designation 20:14/15:6:1:0 of *ERG11*(G1885A) indicates a 20-nucleotide anchor, 14-nucleotide bridge opposite a 15-nucleotide intervening template sequence, and a 7-nucleotide foot composed of 6 nucleotides + 1 interrogating nucleotide positioned at the 3′ end. Similarly, the SuperSelective primer designation of *ERG11*(A434T) 24:13/14:8:1:0 indicates a 24-nucleotide anchor, 13-nucleotide bridge opposite a 14-nucleotide intervening template sequence, and a 9-nucleotide foot composed of 8 nucleotides + 1 interrogating nucleotide positioned at the 3′ end.

**Fig 1:**
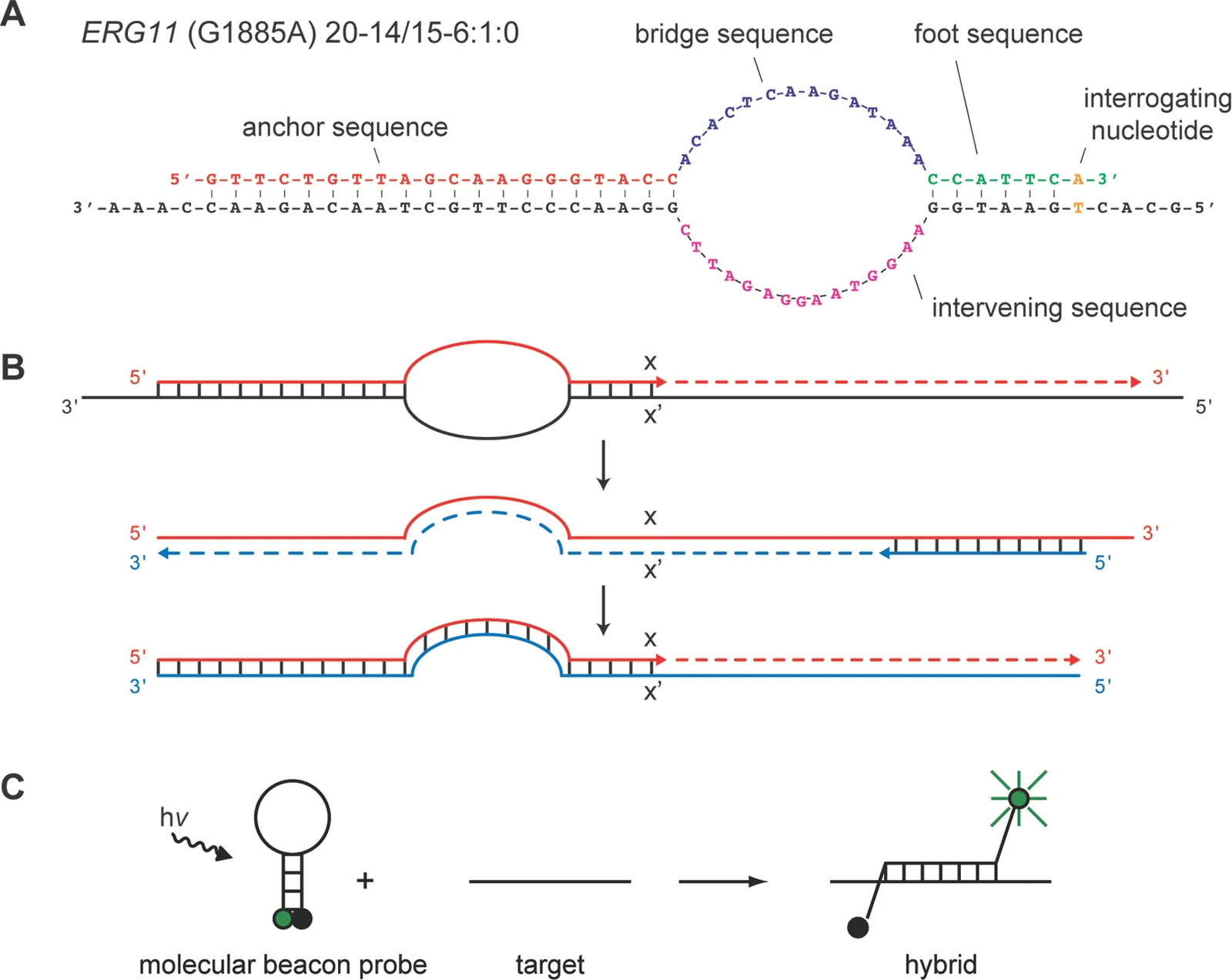
Schematic illustration of SuperSelective primers and molecular beacon design. (**A**) Structure of a SuperSelective primer for detecting *ERG11*(G1885A) mutant sequences in the presence of *ERG11* wild-type sequences. (**B**) Principle of operation of SuperSelective primers: (i) hybridization of the SuperSelective primer with the mutant DNA template fragment, which depends on matching the anchor and the foot sequences (x and x′ denote the SNP), leading to synthesis of a (+) amplicon (red line) from a (−) template; (ii) the (+) amplicon serves as a template for a conventional reverse primer, generating a (−) amplicon (blue-dotted line) with a unique bridge sequence that is complementary to the SuperSelective primer; and (iii) in subsequent thermal cycles, the entire SuperSelective primer sequence is complementary to the (−) amplicon strand, leading to exponential amplification. (**C**) Principal operation of molecular beacons.

**TABLE 1:** Primer sequences used in this study

Primer name	Primer purpose	Primer sequence
CX2475	*ERG11*(A434T) SuperSelective primer forward (24-13/14-8:1:0)	5′ GCTTGTTAGCACTTGACTACTCCCACACCACGAATGATGTTGTTTT 3′
CX2476	*ERG11*(A434T) anchor primer forward	5′ GCTTGTTAGCACTTGACTACTCCC 3′
CX2477	*ERG11*(A434T) reverse primer	5′ GAAATCTTCGCATTCGCTGGTAATC 3′
CX2607	*ERG11*(G1885A) SuperSelective primer forward (20-14/15-6:1:0)	5′ GTTCTGTTAGCAAGGGTACCACACTCAAGATAAACCATTCA 3′
CX2608	*ERG11*(G1885A) anchor primer forward	5′ GTTCTGTTAGCAAGGGTACC 3′
CX2328	*ERG11*(G1885A) reverse primer	5′ AGGGAACTTGGGAACAGCAA 3′

To monitor the PCR amplification signal in real time, we employed the MB probes that bind to the PCR amplicon. In this study, we designed MB probes that bind to *ERG11*(A434T) and *ERG11*(G1885A) regions of *Cn* DNA, respectively (Table 2).

**TABLE 2:** Molecular beacon design for *ERG11* Y145F (A434T) and G484S (G1885A) mutation detection

Molecular beacon	Underlined nucleotides represent arm sequences of the molecular beacon probes
MB_ERG11(A434T)	5′ FAM CGCCGC CAGATCAAGTCCGGTCTTACTACCGA GCGGCG BHQ-1 3′
MB_ERG11(G1885A)	5′ CFR610 CGCGAC CAGGCACAGATGTGTTGGCGAACA GTCGCG BHQ-2 3′

### Determining the sensitivity and specificity of the SSP-PCR

We designed a real-time PCR assay to detect the *ERG11*(A434T) SNP (TAC->TTC) using a SuperSelective primer. To determine SSP-PCR specificity, we first used synthetic 400 bp g-Block templates (an *ERG11* wild-type template and an *ERG11*(A434T) (TAC->TTC) template), allowing us to accurately calculate the template DNA copy number in each PCR assay (Table 3). Using the conventional primer set (CX2476 and CX2477) and SYBR Green to monitor the real-time PCR assay, we observed identical PCR threshold cycles (Ct) from both wild-type *ERG11* and *ERG11*(A434T) mutant templates, confirming the same amount of DNA templates were used (Fig. 2A). Then, we performed real-time PCR assays with the SuperSelective primer CX2475 and the reverse primer CX2477 using the same amount of DNA as a template. We found that the SuperSelective primer set amplified the *ERG11*(A434T) mutant DNA template with a significantly earlier Ct value of 15 cycles for 10^6^ copies compared to the same amount of wild-type (*ERG11*WT) template that was amplified 20 cycles later with a Ct value of 35 (Fig. 2B). Furthermore, the SSP-PCR robustly amplified the *ERG11*(A434T) mutant template, yielding Ct values of 25 and 35 with 10^4^ and 10^2^ copies of DNA template, respectively. In contrast, no amplification was observed with WT templates at the same copy numbers (Fig. 2B).

**Fig 2:**
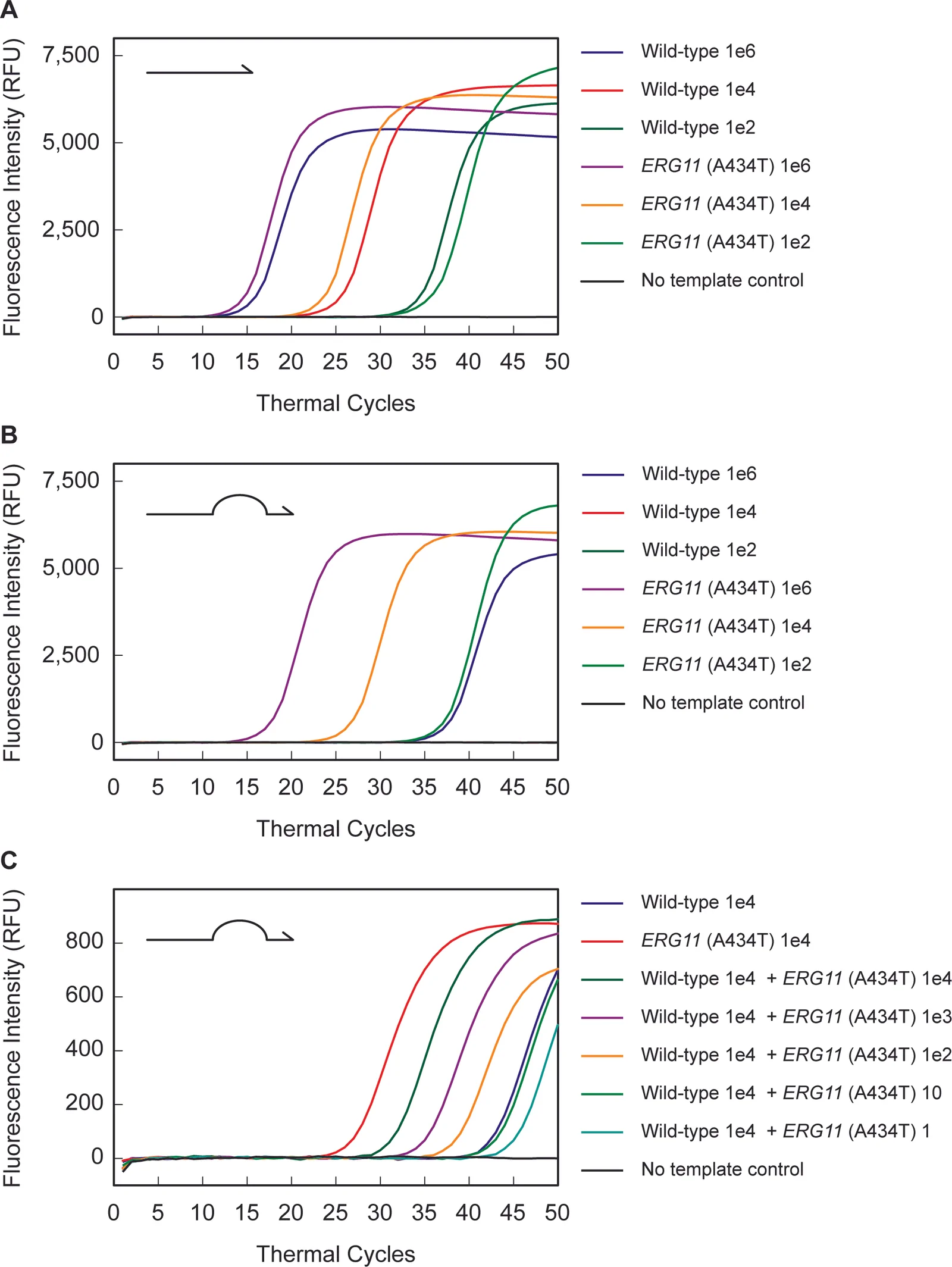
SSP-PCR assay detects *C. neoformans ERG11*(A434T) mutation with high specificity and sensitivity in synthetic gBlock DNA. (**A**) The plot shows real-time PCR amplification curves generated with conventional primers using templates containing either the *ERG11*(A434T) mutation or the wild-type (WT) *ERG11* sequence. These two templates showed comparable amplification at input copy numbers of 10^6^, 10^4^, and 10^2^, confirming equal DNA copy numbers across reactions. (**B**) Real-time PCR amplification curves obtained with SuperSelective primers using templates containing either the *ERG11*(A434T) mutation or the WT sequence. Accordingly, 10^6^, 10^4^, and 10^2^ copies of the mutant template were detected, whereas only the WT template (10^6^ copies) showed significant delayed amplification (Ct 35 cycles), demonstrating the specificity of the SuperSelective primers for *ERG11*(A434T). (**C**) The plot shows real-time amplification curves obtained in the SSP-PCR assay using *ERG11*(A434T) mutant templates in the presence of 10^4^ copies of the WT template. Robust detection of mutant templates at copy numbers of 10^4^, 10^3^, 10^2^, 10, and 1 under these conditions demonstrates the sensitivity of the assay for identifying low-abundance mutants in an excess WT background. No amplification was observed in the no-template control (NTC).

**TABLE 3:** *ERG11* g-Block sequences for Y145F (A434T), G484S (G1885A), and their wild-type (WT) regions^*a*^

g-Block	Sequence
*ERG11* WT (Y145)	GGGTCGAAGGGTTACCGTCGCGCTTGGACCAAAGGGTAACAACCTTTCTTTGGGTGGAAAGA TTTCTCAAGTCTCTGCCGAGGAAGCATACACTGTAAGCTTATGTGCTTCACTGATTTAAGATG G CTTACTTACTGTCGCTTGTTAGCACTTGACTACTCCCGTCTTTGGCAAGGGTGTTGTTT**A**CG ATTGCCCTAATGAGATGCTCATGCAGCAGAAGAAGTTTGTAAGTTAATACCACTCGCAGCTTG ATTCGCAAGCTCATTATTTTACAGATCAAGTCCGGTCTTACTACCGAGTCCCTTCAGTCTTATC CCCCTATGATTACCAGCGAATGCGAAGATTTCTTCACCAAAGAAGTCGGAATTTCTCCCCAGA AGCCTTCTGCCACTCTCGACCT
*ERG11* Y145F (A434T)	GGGTCGAAGGGTTACCGTCGCGCTTGGACCAAAGGGTAACAACCTTTCTTTGGGTGGAAAGA TTTCTCAAGTCTCTGCCGAGGAAGCATACACTGTAAGCTTATGTGCTTCACTGATTTAAGATG GCTTACTTACTGTCGCTTGTTAGCACTTGACTACTCCCGTCTTTGGCAAGGGTGTTGTTT**T**CG ATTGCCCTAATGAGATGCTCATGCAGCAGAAGAAGTTTGTAAGTTAATACCACTCGCAGCTTG ATTCGCAAGCTCATTATTTTACAGATCAAGTCCGGTCTTACTACCGAGTCCCTTCAGTCTTATC CCCCTATGATTACCAGCGAATGCGAAGATTTCTTCACCAAAGAAGTCGGAATTTCTCCCCAGA AGCCTTCTGCCACTCTCGACCT
*ERG11* WT (G484)	ACCCTCGTATCTGGCAAGACGCCAAAGTCTGGAACCCTGCCCGATGGCACGATGAAAAGGG ATTTGCCGCCGCTGCCATGGCTCAATACAGCAAGGCCGAGCAAGTCGATTATGGTTTTGGTT CTGTTAGCAAGGGTACCGAATCTCCTTACCAGCCATTC**G**GTGCCGGCAGGCACAGATGTGTT GGCGAACAATTTGCGTACACTCAACTTTCAACCATCTTCACCTATGTTGTGAGGAATTTCACTT TGAAGCTTGCTGTTCCCAAGTTCCCTGAAACCAATTACCGTGTAAGTCTTCGTGTTCCAATCT ATCACTGGATGGATGTTTTGCTGATCTCCACTCGTGTAGACCATGATTGTCCAACCCAACAAC CCTTTGGTTACTTTCACCCTTCGAAATGCCGAGGTCAAATAG
*ERG11G484S* (G1885A)	ACCCTCGTATCTGGCAAGACGCCAAAGTCTGGAACCCTGCCCGATGGCACGATGAAAAGGG ATTTGCCGCCGCTGCCATGGCTCAATACAGCAAGGCCGAGCAAGTCGATTATGGTTTTGGTT CTGTTAGCAAGGGTACCGAATCTCCTTACCAGCCATTC**A**GTGCCGGCAGGCACAGATGTGTT GGCGAACAATTTGCGTACACTCAACTTTCAACCATCTTCACCTATGTTGTGAGGAATTTCACTT TGAAGCTTGCTGTTCCCAAGTTCCCTGAAACCAATTACCGTGTAAGTCTTCGTGTTCCAATCT ATCACTGGATGGATGTTTTGCTGATCTCCACTCGTGTAGACCATGATTGTCCAACCCAACAAC CCTTTGGTTACTTTCACCCTTCGAAATGCCGAGGTCAAATAG

^*a*^ SNPs are highlighted in bold.

To evaluate the sensitivity of SSP-PCR assays, we mixed wild-type *ERG11* and *ERG11*(A434T) mutant DNA templates where we fixed the concentration of *ERG11*WT at 10^4^ copies and added *ERG11*(A434T) mutant DNA templates at different concentrations: 10^4^, 10^3^, 10^2^, 10, and 1 copies, respectively, and subjected them to real-time PCR using SuperSelective primers integrated with MB probe MB_*ERG11*(A434T) for their detection (Table 2). We detected FAM fluorescence signal for the *ERG11*(A434T) mutant template with Ct values of 29, 32, 36, and 40 cycles for 10^4^, 10^3^, 10^2^, and 10 copies of *ERG11*(A434T) in the presence of 10^4^ copies of *ERG11*WT, respectively. Additionally, SuperSelective primers specifically identified a single copy of the *ERG11*(A434T) mutation DNA with a Ct value of 43 cycles even in the presence of 10^4^ copies of the *ERG11*WT template (Fig. 2C), demonstrating the sensitivity of the SSP-PCR. No WT template amplification was detected, indicating a high PCR specificity. Together, these results confirmed that our SSP-PCR platform enables a robust detection of SNP variants in the presence of abundant WT templates.

### Detection of *ERG11*(A434T) SNP signal in genomic DNA of clinical isolates from *in vitro* cultures and *in vivo* infected mouse lungs

To determine the potential application of the SSP-PCR platform as a future diagnostic method, we tested the PCR assay using genomic DNA from the reference strain H99 and an *mrl1* strain carrying the *ERG11*(A434T) mutation, as well as additional clinical isolates Ugcl236, Ugcl241, Ugcl422, and Ugcl541 (Uganda clinical isolate library from Kristen Nelson’s laboratory (24)). We isolated genomic DNA from fungal strains using the CTAB method and successfully detected the *ERG11*(A434T) mutation in a PCR assay with 50 ng of template DNA in each reaction (Fig. 3A). Using conventional primers CX2476/CX2477, the PCR with both templates had a Ct value of 21-22 cycles, indicating an equal amount of the template DNA in each reaction (Fig. 3A). In our SSP-PCR assay with SuperSelective primer CX2475 and the reverse primer CX2477, we detected the MB signal at 21 cycles with the *mrl1* DNA, while a later Ct value of 35 cycles was observed with all other clinical isolates, including the reference strain H99 template (Fig. 3A), indicating a high *ERG11*(A434T) SNP detection specificity.

**Fig 3:**
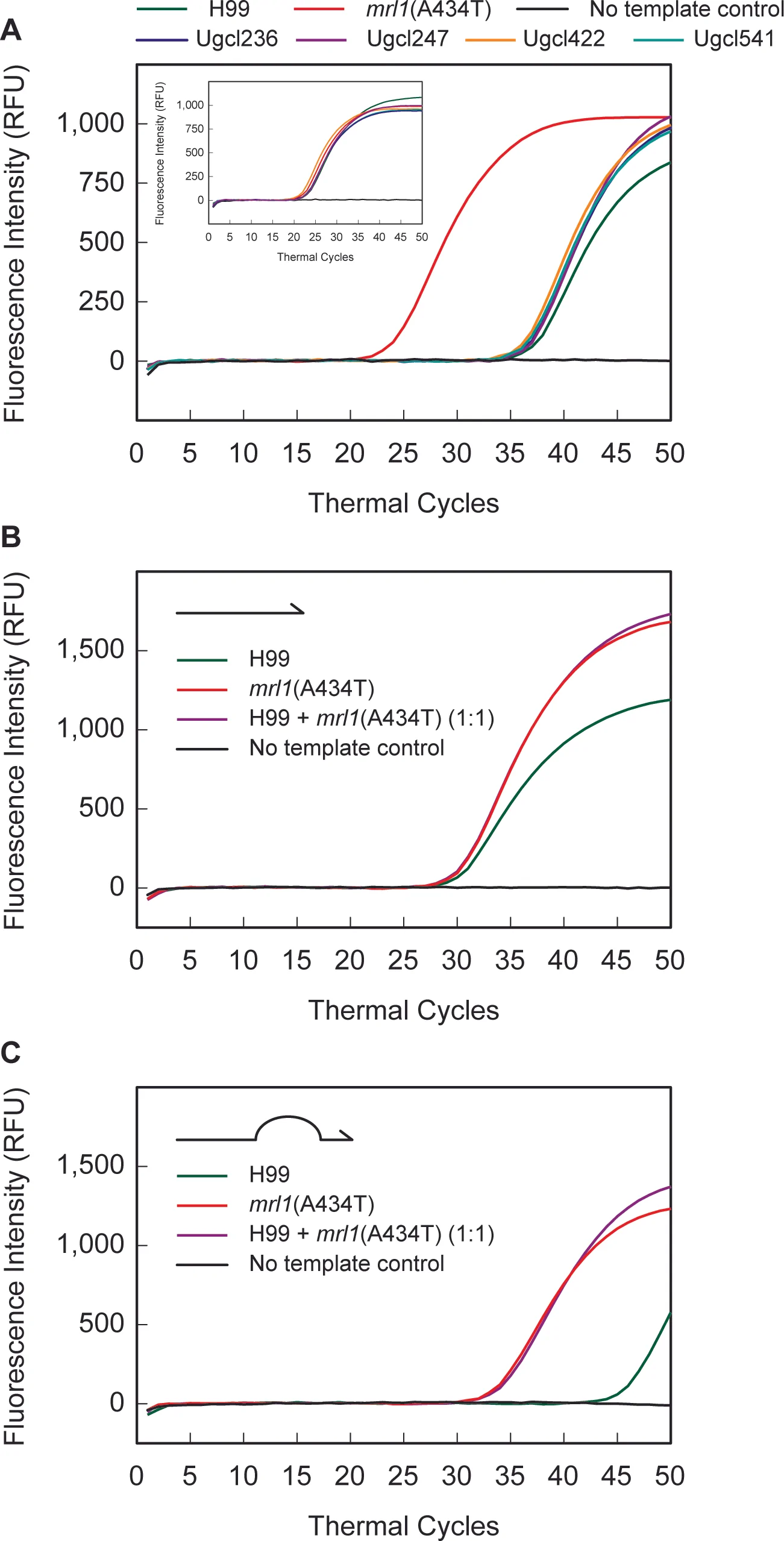
Detection of *C. neoformans* and azole-associated *ERG11*(A434T) mutation in genomic DNA. (**A**) The *ERG11*(A434T)-specific molecular beacon selectively detected the azole resistance-associated mutation in genomic DNA isolated from the *C. neoformans ERG11* (A434T) mutant strain (*mrl1*) at an earlier Ct of 20 cycles compared to genomic DNA isolated from the reference strain WT H99 and other clinical isolates (i.e., Ugcl236, Ugcl247, Ugcl422, and Ugcl541). The inset demonstrates comparable DNA input across samples (Ct 20–21 cycles for all samples), as confirmed by equal amplification with the conventional primers. *In vivo* detection of *C. neoformans* from mouse lungs infected with 10^5^ H99, 10^5^ *mrl1* mutation, or a mixed H99 and *mrl1* cells at a 1:1 ratio. (**B**) Real-time amplification curves generated utilizing the conventional primer set show that all reactions contain approximately the same total number of DNA templates, suggesting similar fungal burden in each sample. (**C**) The plot shows real-time amplification curves for the *mrl1* mutation at Ct 30 cycles, confirming sensitive detection of *mrl1* in *in vivo* infected tissue samples.

Furthermore, to determine the sensitivity of this detection method *in vivo* in infected tissues, we intranasally infected the C57BL/6 mice with 10^5^ cells of either H99 only, a *mrl* mutant strain only, or a mixed inoculum containing both strains at a 1:1 ratio (10^5^:10^5^ copies). Infected lung tissues were isolated at day seven post-infection, and their total genomic DNA was extracted using the CTAB method. Using 45 ng of these total genomic DNAs as templates, our PCR with conventional primer CX2476/CX2477 resulted in identical Ct values (27 cycles), indicating an equal number of fungal DNA copies for each reaction (Fig. 3B). We then performed our SSP-PCR assay using primers CX2475 and the reverse primer CX2477. Our MB probe successfully detected the *ERG11*(A434T) mutation at much earlier PCR cycles, at 31 cycles, than the wild type, which was not amplified until over 45 cycles, indicating a high amplification specificity (Fig. 3C). Overall, our results demonstrate that our SSP-PCR assay in combination with the MB probe can detect the SNP mutations in genomic DNAs isolated from *C. neoformans* strains or their infected lungs.

### Duplex detection of *ERG11*(A434T) and *ERG11*(G1885A) mutations

Clinical specimens may harbor mixed *Cryptococcus* populations that contain multiple azole resistance alleles. To assess whether our SSP-PCR platform can detect two mutations simultaneously, we developed a duplex assay as a proof of concept. In line with this, we designed a SuperSelective primer assay for another known azole resistance-inducing mutation, *ERG11*(G1885A). Before testing the duplex assay, we performed a simplex assay to determine the amplification efficiency. To this end, we diluted the g-Block templates of *ERG11*(G1885A) and *ERG11*(A434T) to 10^6^, 10^5^, 10^4^, 10^3^, 10^2^, 10, and 1 copies individually and subjected them to SuperSelective primer PCR assays. As expected, SuperSelective primers CX2475 and CX2477 detected *ERG11*(A434T) in FAM with Ct values of 15, 18, 23, 26, 29, 33, 37, and 41 cycles for 10^6^, 10^5^, 10^4^, 10^3^, 10^2^, 10, and 1 copies (Fig. 4A). We detected *ERG11*(G1885A) in Cal fluorescence Red 610 (CFR610) using SuperSelective primer CX2607 (20-14/15-6:1:0) and the reverse primer CX2328, with Ct values of 23, 26, 30, 34, 38, 44, and 47 cycles for 10^6^, 10^5^, 10^4^, 10^3^, 10^2^, 10, and 1 copies (Fig. 4B).

**Fig 4:**
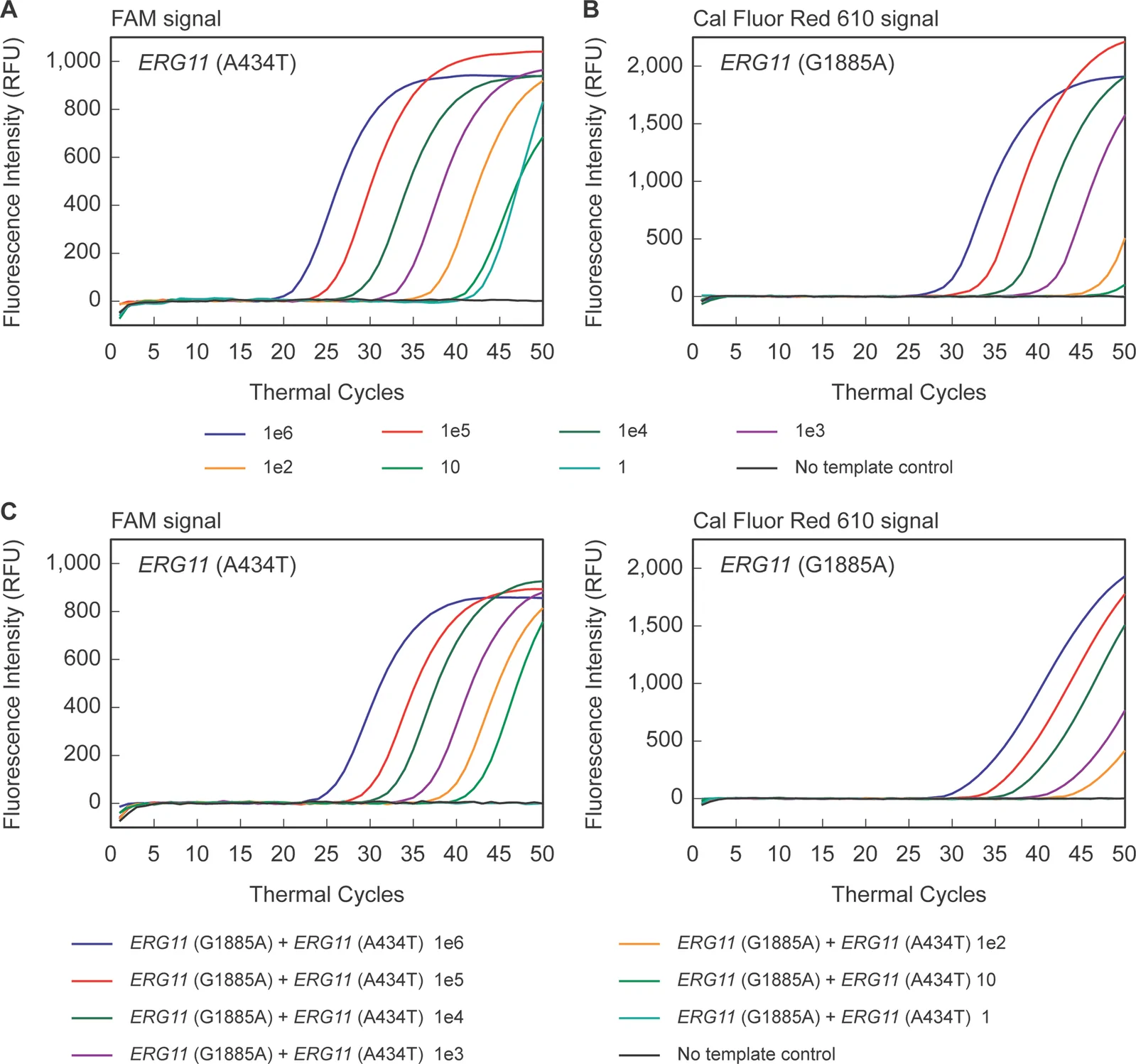
Concurrent detection of *ERG11*(A434T) and *ERG11*(G1885A) mutations in a duplex reaction using SSP-PCR. A simplex assay individually detects two azole resistance-associated SNPs—*ERG11*(A434T) and *ERG11*(G1885A)—in two different fluorophore channels. At the concentrations of 10^6^, 10^5^, 10^4^, 10^3^, 10^2^, 10, and 1 copies, (**A**) FAM-channel amplification curves show specific detection of the *ERG11*(A434T) targets, and (**B**) Cal Fluor Red 610 (CFR610)-channel amplification curves show specific detection of *ERG11*(G1885A) targets. (**C**) For a duplex SSP-PCR assay, varying concentrations (10^6^, 10^5^, 10^4^, 10^3^, 10^2^, 10, and 1 copies) of *ERG11*(G1885A) templates were combined with *ERG11*(A434T) templates at (10^6^, 10^5^, 10^4^, 10^3^, 10^2^, 10, and 1 copies) concentration. FAM-channel amplification curves show specific detection of the *ERG11*(A434T) target, while CFR610-channel amplification curves show specific detection of the *ERG11*(G1885A) target. Each SuperSelective primer-probe set selectively detected its intended target.

For the duplex assay, we combined both the mutant DNA g-Blocks with the same amount of template DNAs at varying concentrations, ranging from *ERG11*(G1885A) (10^6^, 10^5^, 10^4^, 10^3^, 10^2^, 10, and 1 copies) + *ERG11*(A434T) (10^6^, 10^5^, 10^4^, 10^3^, 10^2^, 10, and 1 copies). As expected, each primer set specifically detected its intended mutant target without loss of specificity. Using these pooled samples, *ERG11*(G1885A) generated a fluorescent signal in the Cal Fluor Red 610 channel with a Ct of 28, 32, 35, 39, and 42 cycles for *ERG11*(G1885A) present at concentrations of 10^6^, 10^5^, 10^4^, 10^3^, and 10^2^, respectively, in the background of *ERG11*(A434T) at the same concentrations. Furthermore, *ERG11*(A434T) generated a FAM fluorescent signal with Ct values of 22, 27, 30, 33, 36, and 37 cycles at concentrations of 10^6^, 10^5^, 10^4^, 10^3^, 10^2^, and 10 copies, respectively. Importantly, the assay detected as few as 10^2^ copies of *ERG11*(G1885A) and 10 copies of *ERG11*(A434T), demonstrating the sensitivity of the duplex format (Fig. 4C). These results demonstrate that the SSP-PCR platform can selectively and sensitively detect two resistance-associated SNPs in a single tube reaction, highlighting its potential as a multiplex assay for high-throughput screening.

## DISCUSSION

Cryptococcosis is one of the leading causes of death in immunocompromised patients, imposing a disproportionate public health burden in resource-limited settings. Early diagnosis is critical to guiding proper therapeutic interventions in reducing cryptococcosis-associated morbidity and mortality (5). However, the current diagnosis for cryptococcosis is inadequate. Even in a closely monitored high-risk population, confirmation of cryptococcal meningitis takes 2–3 weeks from symptom onset (25). Diagnosis is mainly based on a combination of histopathology, direct microscopy, fungal culture, and/or a positive *Cryptococcus* antigen test (26). Antigen-based assays, like detection of polysaccharide capsule in serum and cerebrospinal fluid by enzyme-linked immunosorbent assays (ELISA), latex flow assay (LFA), or latex agglutination, are highly sensitive yet less effective for the detection of pulmonary infection (5) and are the current clinical gold standard for cryptococcal diagnosis. Moreover, PCR-based methods have been developed to either identify all *Cryptococcus* species based on the fungal internal transcribed spacer (*ITS*) region or distinguish different species with species-specific primers targeting the fungal mitochondrial cytochrome b gene (cyt b) sequence (27, 28). However, none of these currently available diagnostic methods can identify cryptococcal antifungal drug resistance, including heteroresistance in a clinical setting.

Chromosomal duplication resulting in aneuploidy is most frequently observed in patients with cryptococcal meningitis undergoing FLC monotherapy (8, 29–31). Rapid identification of antifungal resistance remains as a significant diagnostic challenge in pulmonary cryptococcosis, particularly when the resistance arises from SNPs within genetically heterogeneous or low-burden clinical specimens. Although sequencing-based approaches, such as next-generation sequencing, can detect SNPs, they are expensive, time-consuming, and require specialized equipment and experienced personnel. Conventional PCR assays with differently colored TaqMan probes or molecular beacons can be used to distinguish mutants from wild type. However, these assays utilize outside primers that amplify both mutant and their closely related wild-type sequences, resulting in weak amplification of the mutant target and limiting the ability to detect rare mutant sequences in the background of abundant wild-type DNA (18, 20). In hopes of overcoming these limitations, SSP-PCR assays capable of discriminating single-base changes in mixed genetic backgrounds without amplifying the wild-type template offer a promising alternative (18, 20).

In this study, we developed an SSP-PCR platform capable of detecting two known azole resistance-associated single-nucleotide polymorphisms in the *ERG11* gene of *C. neoformans*. The ability to detect the *ERG11*(A434T) mutant allele in synthetic g-Block templates despite the presence of wild-type sequence underscores the single-base specificity of this assay. This performance extended to genomic DNA from a hyper-resistant isolate, *mrl1*, that contains this mutation and, importantly, to samples derived from a murine infection model in which mice were infected with either H99, *mrl1*, or a combination of H99 and *mrl1*. In the mixed-infection setting, the platform successfully identified the *mrl1*-associated mutation, demonstrating its ability to detect mutant alleles in the presence of an abundant wild-type background *in vivo*. Additionally, coupling SuperSelective primers with molecular beacon probes enabled simultaneous detection of two azole resistance-associated mutations, *ERG11*(A434T) and *ERG11*(G1885A), in a single reaction. These findings highlight the promise of this platform as a rapid molecular diagnostic strategy for detecting resistance-associated alleles, particularly in heterogeneous samples containing both susceptible and resistant populations.

SSP-PCR assays, by virtue of their design (Fig. 1A), exponentially amplify the mutant DNA template, even in the presence of their closely related wild-type DNA template. During the PCR annealing, the SuperSelective primer’s foot sequence forms stable foot-target hybrids that persist for longer times, which is sufficient to permit a polymerase to initiate the synthesis of an amplicon, while the mismatched foot-target hybrids are so short-lived that they very rarely initiate the synthesis of an amplicon. Since the foot sequence is very short, it results in a large enthalpic difference between binding of the primer to the perfectly complementary mutant target sequence and to the mismatched wild-type target. Adding tetramethylammonium chloride (TMAC) in the PCR assay further enhances this by stabilizing the matched mutant hybrids while destabilizing the mismatched wild-type target (32). Combining the specificity of SuperSelective primers with molecular beacons results in a versatile diagnostic platform that has been used to detect drug resistance-associated mutations in several different infectious diseases and cancer (18, 19). A clear precedent of the SSP-PCR assay demonstrated whether a patient is infected with *Mycobacterium tuberculosis*, the bacterial load, and whether the strain is resistant to the antibiotic rifampin all within 2 h using the patient’s sputum (33). A recent study demonstrated an alternative design for SuperSelective primers, known as the Self-Competitive Fishing Primer, that combined SuperSelective primers with wild-type blocking oligonucleotides to prevent amplification of wild-type DNA while enhancing mutant DNA detection in endometrial cancer, offering a practical alternative to next-generation sequencing (34).

MBs are added prior to amplification; they fluoresce in real time in the presence of their targets, enabling quantitative measurement of the starting template while minimizing background noise from primer dimers, which commonly affect the PCR signal specificity. MBs have been employed to detect *ERG11* mutations in *Candida auris* (35) and FKS-associated echinocandin resistance in *Candida auris* (35), *C. albicans* (36), and *C. glabrata* (37). However, a limitation of MBs or other hybridization probe-based SNP detection methods is that they cannot detect a rare mutant population when it is less than 5–10% of the total population. When combined with SuperSelective primers, this configuration enhances SNP discrimination within mixed-allele pools. Thus, their structural and thermodynamic properties make them suitable candidates for SNP-level diagnostics (18–20, 32, 33).

Despite these strengths, our current study has a few limitations. First, our method is only suitable to detect mutation-induced drug resistance, such as SNPs in *ERG11*. Other methods will be required to detect drug resistance due to other mechanisms, such as *ERG11* overexpression or enhanced efflux pumps, etc. Second, unlike bedside cryptococcal antigen detection LFA assays, our platform requires genomic DNA isolation, which might limit immediate point-of-care implementation. However, this limitation could be alleviated by developing quick DNA isolation assays. Third, although the assay was evaluated using genomic DNA isolated from an infected mouse lung tissue, this does not fully encompass the complexity of human clinical specimens. Hence, our future studies will evaluate whether the defined mutant templates spiked into human genomic DNA affect the sensitivity and specificity of the assay. Our PCR validation was performed under experimentally heterogeneous conditions using several clinical isolates rather than a large-scale clinical cohort to establish diagnostic sensitivity and specificity. Additionally, the current assay targets only a few azole resistance-associated mutations, whereas clinical resistance may arise from multiple mutations within *ERG11* or other loci. However, the combination of SuperSelective primers with MB technology enables us to design multiple mutant-specific SuperSelective primers with different fluorophores for a multiplex panel in a single tube. Therefore, our future work will focus on developing a multiplex panel capable of simultaneously detecting multiple azole resistance mutations within a single reaction, thereby enhancing the clinical utility and scalability.

In conclusion, we have established a SuperSelective primer-based PCR platform for the selective detection of azole resistance-associated *ERG11* mutations: *ERG11*(A434T) and *ERG11*(G484S) in *C. neoformans*. The assay discriminated mutant templates from the WT sequences across synthetic and genomic DNA backgrounds and enabled simultaneous detection of two resistance-associated SNPs in a duplex format. To our knowledge, this is the first molecular assay to distinguish mutant targets under experimentally heterogeneous conditions without cross-reactivity, even *in in* vivo-infected tissues. We understand that azole resistance can arise through multiple mechanisms beyond the mutations examined here; our assay serves as a highly selective drug-resistance detection platform rather than a comprehensive diagnostic for antifungal resistance. With increased understanding of the azole resistance mechanisms and identification of additional resistance-associated mutations in *ERG11*, our current proof-of-concept method may be further expanded to a multiplex platform that allows us to detect more resistance-associated targets in a single PCR reaction. With a detailed, resistance-associated mutation map, together with validation in larger clinical cohorts, this method could support the development of a multiplex panel and may complement existing diagnostic strategies to improve clinical outcomes. Additionally, this platform can also be expanded for the detection of other drug-resistant fungal pathogens to advance therapeutic efficacy in clinical settings.

## MATERIALS AND METHODS

### Strains and growth conditions

All fungal strains were streaked onto YPD agar plates and grown in YPD liquid medium at 30°C overnight with shaking. The following fungal strains were used in the study: *C. neoformans* reference strain H99 and *C. neoformans* isolate carrying *ERG11* Y145F (A434T) mutation (*mrl),* a clinical isolate gifted by Dr. Jun Kwon-Chung from NIH, *C. neoformans* clinical isolates Ugcl236, Ugcl241, Ugcl422, and Ugcl541 (Uganda clinical isolate library) gifted by Dr. Kristen Nielson. Chemicals and primers were purchased from Sigma-Aldrich (St. Louis, MO) unless specified.

### Isolation of genomic DNA from infected mouse lung using CTAB method

Briefly, 0.6 mL of lung homogenate was transferred into the 2 mL screwcap tubes, and 0.7 mL of autoclaved ddH_2_O was added. The tubes were placed at 4°C overnight to lyse the mammalian cells. The next day, the tubes were centrifuged at 7,000 rpm for 10 min, and the supernatant was discarded. The cells were washed again with autoclaved ddH_2_O. The samples were resuspended in 8 M urea buffer (8 M urea, 0.5 M NaCl, 20 mM Tris, 20 mM EDTA, and 2% SDS) for 4 h at room temperature with gentle agitation. Cell pellets collected by 5 min centrifugation at 2,000 rpm, and 0.4 mL of acid-wash beads was added to the tube along with the 0.4 mL of CTAB buffer (1 M Tris pH 7.5, 5 M NaCl, 0.5 M EDTA, CTAB [Sigma-Aldrich, St. Louis, MO] M7635) and β-mercaptoethanol (14 M), followed by bead-beat for 3–5 min. Additional CTAB buffer was added to reach ~600 µL, and the tubes were incubated at 65°C on a heat block before cooling to room temperature. An equal amount of chloroform was added, gently mixed, and centrifuged at 8,000 rpm for 10 min. The clear aqueous phase was transferred to a fresh tube; an equal volume of isopropanol was added. Incubated 30 mins at −20°C, followed by centrifugation at 10,000 rpm for 10 mins. Supernatant was discarded. The pellet was washed with 70% ethanol and air-dried. The pellet was dissolved in TE buffer.

### Mouse infection

A group of three C57BL/6 mice (Jackson Laboratory, Bar Harbor, ME) was intranasally infected with 10^5^ yeast cells per mouse of *C. neoformans* H99, the *mrl* strain containing *ERG11*(A434T) mutation, or in a 1:1 combination [*ERG11*(A434T):*ERG11*WT], respectively. Mice were sacrificed 7 days post-infection, and their lungs were harvested for the SSP-PCR assay. The mouse lungs were homogenized in 1× PBS using a homogenizer, and total DNA was extracted by the CTAB method as described above.

### Primers, g-Blocks, and molecular beacons

SuperSelective primer sequences were examined with the Mfold web server and the OligoAnalyzer computer program (Integrated DNA Technologies, Coralville, IA) to ensure that they do not form hairpin structures, self-dimers, or heterodimers with conventional reverse primers. The primers were purchased from Integrated DNA Technologies or Sigma-Aldrich (St. Louis, MO), and the MB probes for detecting the amplicons were purchased from Biosearch Technologies (Petaluma, CA). The synthetic g-Block DNAs of *C. neoformans* azole-associated *ERG11* mutations: Y145F(A434T) and G484S(G1885A) were purchased from Integrated DNA Technologies (Table 3).

### Real-time PCR assays

Monoplex real-time PCR assays were performed in 25 μL volumes containing 200 nM of each forward (SuperSelective or conventional) and reverse primer, and 1.5 mM MgCl2, 20 mM of TMAC, Taq 2× MasterMix (Platinum Hot start PCR 2×), and 1× SYBR Green were purchased from Thermo Fischer Scientific (Waltham, MA) and added to monitor template amplification (20 μL reaction mix + 5 μL of template).

Duplex real-time PCR assays were carried out in 25 μL volumes containing 200 nM of each forward (SuperSelective or conventional) and reverse primer, 1.5 mM MgCl2 (Invitrogen), 20 mM of TMAC, Taq 2× MM (Platinum Hot start PCR 2×), and 200 nM of each MB for monitoring amplicon generation during the annealing stage of each thermal cycle (20 μL reaction mix + 5 μL of template).

All amplifications were carried out in 200 μL white polypropylene PCR tubes (USA Scientific, Ocala, FL) in a Bio-Rad CFX96 Detection System (Bio-Rad Laboratories, Hercules, CA). All reactions were carried out using the following PCR cycling conditions: hot start at 95°C for 2 min to activate the polymerase, followed by 50 cycles of 95°C for 15 s denaturation and 60°C for 20 s annealing, with fluorescence monitoring.

## Data Availability

Data are provided within the manuscript or supplemental material. The strains and other materials for the current study are available from the corresponding authors on reasonable request.
